# High Hydrostatic Pressure as a Tool to Reduce Formation of Biogenic Amines in Artisanal Spanish Cheeses

**DOI:** 10.3390/foods7090137

**Published:** 2018-08-30

**Authors:** Diana Espinosa-Pesqueira, Maria Manuela Hernández-Herrero, Artur X. Roig-Sagués

**Affiliations:** CIRTTA-Departament de Ciència Animal i dels Aliments, Universitat Autònoma de Barcelona, Travessera dels Turons S/N, 08193 Barcelona, Spain; diespe@gmail.com (D.E.-P.); manuela.hernandez@uab.cat (M.M.H.-H.)

**Keywords:** biogenic amines, cheese, high hydrostatic pressure

## Abstract

Two artisanal varieties of cheese made in Spain, one made of ewes’ raw milk and the other of goats’ raw milk were selected to evaluate the effect of a high hydrostatic pressure (HHP) treatment at 400 MPa during 10 min at 2 °C on the formation of biogenic amines (BA). These conditions were applied at the beginning of the ripening (before the 5th day; HHP1) and in the case of ewes’ milk cheeses also after 15th days (HHP15). BA formation was greatly influenced by HHP treatments in both types of cheese. HHP1 treatments significantly reduced the amounts of BA after ripening, being tyramine and putrescine the most affected BA in goats’ milk cheeses and tyramine and cadaverine in ewes’ milk cheeses. The BA reduction in the HHP1 samples could be explained by the significant decrease in microbiological counts, especially in the LAB, enteroccocci and enterobacteria groups at the beginning of ripening. The proteolysis in these samples was also affected reducing the amount of free amino acids. Although proteolysis in ewes’ milk cheeses HHP15 was similar than in control samples a reduction of BA was observed probably because the decrease caused on microbial counts.

## 1. Introduction

Biogenic amines (BA) are basic nitrogenous compounds formed in different foodstuffs due to the microbial decarboxylation of amino acids. The kind and the amount of BA formed depends on the decarboxylase capability of the bacterial strains present, the availability of substrate amino acids and the physicochemical properties of the matrix [1]. Some aromatic BA, such as histamine (HIS), tyramine (TY), β-phenylethylamine (PHE) and tryptamine (TR), have psychoactive and vasoactive properties that may cause food poisoning when present in foodstuffs, while the diamines putrescine (PU) and cadaverine (CA) can boost the toxic action of aromatic BA [2,3]. Outbreaks caused by HIS are frequent, although not always correctly diagnosed or declared. It causes intoxication—called “histamine food poisoning” or “scombroid food poisoning”—since it is frequently associated with consumption of scombroid fish, especially tuna fish and mackerel [4]. After fish, cheese is the most commonly implicated food product associated with histamine poisoning and outbreaks have been associated with cheeses made from both raw and pasteurized milk [5]. The HIS concentrations in cheeses that were implicated in outbreaks ranged between 850 to 1870 mg/kg [6]. Tyramine is another BA associated with food-borne poisoning, being one of the causative agents of the so called “cheese reaction” [7], causing migraine, headache and, in extreme cases, hypertensive breakdown [5,8,9]. The seriousness of any BA food-borne poisoning depends on the ingested dose and the sensitiveness of the consumer (genetic or acquired) [3,10].

During cheese manufacture several factors may contribute to the accumulation of toxic amounts of BA. Good manufacturing practices to minimize the occurrence of BA-producing microorganisms in raw materials, pasteurization of milk or addition of BA-non-producing starter cultures have been suggested as BA risk mitigation options. Although it has been described that milk pasteurization reduces the level of decarboxylase-positive bacteria, later contamination of milk and curd during cheese manufacturing by decarboxylating bacteria and their subsequent growth and metabolic activity during cheese ripening usually results in BA build up [10,11,12,13].

High hydrostatic pressure (HHP) is non-thermal processing method used to extend shelf-life of foods. This induces morphological changes and inhibition of enzymes and genetic mechanisms of microorganism [14]. HHP offers the advantage that can be applied after cheese manipulation is over and no further contamination of curd is expected. The effect of HHP treatments has been evaluated in different kind of cheeses made from cows’ milk [15], ewes’ milk [16] and goats’ milk [17,18], being able to eliminate most of the pathogenic bacteria associated with this product but it may also be useful to eliminate decarboxylating bacteria and avoid BA formation during cheese ripening. Calzada et al. [19] evaluated HHP treatments up to 600 MPa at different ripening stages to control the excessive proteolysis and BA formation on blue veined cheese, observing a lower concentration of TY. In another study [20], it was observed that HHP treatments significantly reduced the BA build up when applied on days 21 and 35 of ripening in “Torta del Casar” type cheese. This kind of cheese is made of raw milk and vegetable rennet, which causes a strong proteolytic activity and leads to extensive caseins breakdown in cheese matrix. Nevertheless, much less information was found about the HHP effect on the BA formation in other type of cheeses made of pressed paste.

The main objective of this survey was to evaluate HHP processing to reduce BA formation in two varieties of artisanal Spanish cheese made of raw milk from ewe and goat. Treatments were applied at different stages of ripening and the consequences of these treatments on the BA formation and proteolytic activity were evaluated during the ripening.

## 2. Materials and Methods

### 2.1. Cheese Manufacturing

Two types of artisanal ripened cheeses elaborated in Spain were studied in this work, both made with starter culture, enzymatic curd and pressed paste. The first one was produced from goats’ raw milk in the region of Catalonia, northeast of Spain, and the second was made from ewes’ raw milk in Castilla y León, central Spain. Three independent batches of each type of cheese were produced following the usual manufacturing procedures used by the manufacturers.

### 2.2. High-Hydrostatic Pressure (HHP) Treatments

HHP treatments were performed at 400 MPa for 10 min at a temperature of 2 °C using an Alstom HHP equipment (Alstom, Nantes, France) with a 2 L pressure chamber. A mixture of alcohol and water (1:9) was used into the chamber. The pressurization rate and depressurization time were 268 MPa/min and 55 s, respectively. Before processing, goats’ milk cheese samples were separated in two batches: samples not HHP treated (Control samples) and samples HHP treated before the 5th day of ripening (HHP1). In the case of ewes’ milk cheese samples a third batch for samples that were treated after 15 days of ripening (HHP15) was included. In all cases a portion of about 8.0 cm diameter was obtained and shaped to adequate it to the diameter of the cylinder of the HHP equipment and then vacuum packaged. After the HHP treatments, samples were deprived of the plastic bag and kept into a ripening chamber at 14 °C and 88% of relative humidity to continue with the ripening process for 60 days. Analyses of cheeses were performed at the 5th, 15th, 30th, 45th and 60th day of ripening.

### 2.3. Microbiological Analysis of Cheeses

Ten grams of each sample were homogenized in 90 mL of sterile Buffered Peptone Water (Oxoid, Basingstoke, Hampshire, UK) with a paddle blender (BagMixer, Interscience, France). Counts of *Lactococcus* spp. were made on M-17 agar (Oxoid) supplemented with a bacteriological grade lactose solution (5 g/L, Oxoid) and incubated at 30 °C for 48 h; Lactobacilli were determined on Man Rogose Sharpe agar (MRS, Oxoid) and incubated at 30 °C for 48 h; Enterococci were enumerated using KF *Streptococcus* Agar (Oxoid) supplemented with 2,3,5-trifeniltetrazolium chloride solution 1% (Oxoid) and incubated at 37 °C for 48 h; enumeration of *Enterobactericeae* was performed on Violet Red Bile Glucose Agar (VRBG, Oxoid) and counts of *Escherichia coli* was made on the chromogenic selective media Coli ID (BioMérieux, Marcy l’Etoile, France) and incubated at 30 °C for 24 h; *Staphylococcus aureus* counts were determined on Bair Parker Agar supplemented with rabbit plasma fibrinogen (BP-RPF agar, BioMérieux, Marcy L’Etoile, France) and incubated at 37 °C for 24–48 h.

### 2.4. Assessment of Proteolysis Activity on Cheeses

Water Soluble Extracts (WSE) of cheese were prepared according to the method described by Kuchroo and Fox [21]. From the WSE adjusted at pH 4.6, Water-Soluble Nitrogen (*WSN*) fraction was obtained and determined by the Dumas combustion method [22]. The nitrogen content of the *WSN* fraction was expressed as a percentage of Total Nitrogen (*TN*), described as the Ripening Index (*RI*), according to the formula:

(1) RI=(WSN/TN)×100

The measurement of the amino group content was determined on WSE by the Trinitrobenzensulphonic Acid method (TNBS) according to the procedure described by Hernández-Herrero et al. [23]. Results were expressed as mg of L-Leucine per g of cheese. The total Free Amino Acids (FAA) content was determined on WSE by the cadmium-ninhydrin method described by Folkertsmaa and Fox [24] and the results expressed as mg of l-Leucine per g of cheese.

### 2.5. Determination of Biogenic Amines in Cheese

The RP-HPLC method described by Eerola et al. [25] and modified by Roig Sagués et al. [12] was used to determine the BA content in cheese samples. The BA determined were β-phenylethylamine (PHE), tryptamine (TR), putrescine (PU), cadaverine (CA), histamine (HIS), tyrosine (TY), spermidine (SD) and spermine (SM).

### 2.6. Statistical Analysis

Analysis of variance (ANOVA) was performed on all data from each batch and treatment of goats’ and ewes’ milk cheeses at different ripening stages. Comparisons of mean values of physicochemical, microbiological and BA were followed by Duncan test with significance level set on *p* < 0.05. Comparisons of mean values of proteolysis indexes were performed using the Student-Newman-Keuls test with the significance level set at *p* < 0.05. All tests were performed with the SPSS for windows (v.15.01) program (SPSS Inc., Chicago, IL, USA).

## 3. Results and Discussion

### 3.1. Effect of HHP Treatment on Microbial Counts

Table 1 and Table 2 shows the growth of the microbial groups counts along the ripening process in the goats’ and ewes’ cheeses, respectively, in both control and HHP processed samples.

In control samples (non HHP-treated) of both types of cheese, Lactoccocci was the main microbial group at the beginning of the ripening, showing counts above 8 log_10_ CFU/g, probably because strains of *Lactococcus lactis* were added as the starter culture, while lactobacilli counts were significantly lower at the beginning of the ripening, although their counts rose as the ripening progressed, achieving similar counts to lactococci. Enterococci counts remained steady at a level about 6 log_10_ CFU/g at the beginning of the process, showing a slight decrease around the 15th and the 30th day in the goats’ and ewes’ cheeses, respectively. *S. aureus*, enterobacteria and *E. coli* counts decreased during the ripening until becoming undetectable in most of the samples. These results are in agreement with those reported by other authors in other varieties of cured cheeses [26,27,28,29,30].

HHP treatments reduced significantly the Lactic Acid Bacteria (LAB) counts. In HHP1 treated samples, lactoccocci counts decreased 2.2 log_10_ CFU/g and 1.5 log_10_ CFU/g in the goats’ and ewes’ milk cheeses, respectively. In the case of lactobacilli mean reductions were 3.41 log_10_ CFU/g and 1.03 log_10_ CFU/g. However, these counts recovered along the ripening process and no statistical differences were observed at the end of the work with respect to control samples. Ewes’ milk cheeses HHP15 showed a reduction of about 1.91 log_10_ CFU/g and 1.33 log_10_ CFU/g for lactoccocci and lactobacilli counts, respectively. In that case the subsequent recovery of these counts was not so clear and they did not achieve the same counts than control samples. Novella-Rodríguez et al. [31] also observed that starter counts were reduced about 2 log_10_ CFU/g in goats’ milk cheeses as a consequence of HHP, although a subsequent increase was found during ripening. Rynne et al. [32] reported significant reductions of about 1.5 log_10_ CFU/g in starter and non-starter LAB in cheddar cheese treated at 400 MPa on the first day of ripening. In ovine milk ripened cheeses treated on day 1 and 15 at 300 MPa and 400 MPa proved to cause similar reductions although recovery of LAB counts was observed only in samples treated 1st day. HHP treatments also affected significantly (*p* < 0.05) the counts of enteroccocci in both type of cheeses in either HHP1 and HHP15 samples, being unable to recover the initial counts in any case. Arqués et al. [27] reported a reduction of 2.68 log_10_ CFU/g in enteroccocci counts when a treatment of 400 MPa for 10 min at 10 °C was applied on the 2nd day of ripening to “La Serena” cheeses, remaining constant during the rest of the ripening. Although high counts of enteroccoci have been associated with the unhygienic processing of cheese, their presence is also considered important for the development of the typical aroma and flavour of traditional Mediterranean cheeses. Their counts may range from 10^4^ to 10^6^ CFU/g in curds and 10^5^ to 10^7^ CFU/g in ripened cheeses [33]. In the case of *S. aureus* HHP1 treatments also caused significant reductions in both kind of cheeses and HHP15 in ewes’ milk cheeses, becoming undetectable in most cases at the last day of the work. Nevertheless, the initial counts were already quite low. Similar results were reported in “La Serena” cheeses treated at 400 MPa on the 2nd day of ripening [27] and in Cheddar cheeses and their slurries treated at 400 MPa for 20 min at 20 °C [34]. *S. aureus* has been described as one of the most HHP resistant non-sporulated bacteria. López-Pedemonte et al. [35] described that at least 500 MPa HHP treatments would be necessary to achieve reductions of about 6 log_10_ CFU/g.

HHP treatments showed to be very effective reducing Gram-negative bacteria except for HHP1 in goats’ milk cheese samples, where a slight growth was noted on the 15th day probably due to a possible recovery of the sub lethal injured cells after the HHP treatment. However, cheese ripening conditions, with low pH, increasing salt concentration and presence of LAB, made difficult this recovery to consolidate and no positive counts were detected later at the 30th and 45th days of ripening. Juan et al. [28] also described reductions above 3 log_10_ CFU/g of enterobacteria in ewes’ milk cheeses after a 400 MPa treatment applied the 1st and the 15th day of ripening. Initial counts of *E. coli* were significantly lower than enterobacteria. O’Reilly et al. [34], Capellas et al. [36] and De Lamo-Castellvi et al. [37] have previously pointed out the greatest sensitivity of *E. coli* to HHP in cheeses with reductions above 6 log_10_ CFU/g after HHP treatments at 400 MPa.

### 3.2. Effect of HHP on the Proteolysis of Cheeses

The proteolytic activity parameters measured in the goats’ and ewes’ cheeses are presented in Table 3 and Table 4, respectively.

Significant differences between control and HHP1 samples in TNBS were observed from the 45th to the 60th days of ripening in goats’ milk cheeses, while the differences in FAA content were mainly noted from day 30 to 60. In both parameters control samples showed the highest values. The ratio of RI displayed a different trend where an increment of around two times was observed during the first 15 days period in control and HHP-treated samples but after this point the proteolysis rate became slower. In the ewes' milk cheeses, an increment on the three-proteolysis index evaluated was observed during the ripening period (Table 4). In the ewes’ milk cheeses HHP15 samples presented slightly higher values than control samples, especially in TNBS, although no significant differences were observed at the end of the ripening. Although the HHP1 samples showed an intense proteolysis in the first 30 days of ripening, after this time a decrease on the rate was noticed, obtaining a considerable reduction on the three proteolysis index values at the 60th day, reflecting that pressure treatment caused a deceleration in the rate of proteolysis (Table 4).

RI values indicated that proteolysis was more intense during the first 15 days of ripening in the goats’ and ewes’ cheeses in control and HHP treated samples. This proteolytic activity was probably caused by milk and rennet proteinases, being not so clear the role of microbial proteinases. On fact, *WSN* is produced mainly by the rennet and to a lesser extent by plasmin or cellular proteinases, whereas starter peptidases are primarily responsible for the formation of small peptides and free amino acids [38]. Messens et al. [39] observed that chymosin and plasmin activity in “Gouda” cheese was not influenced by pressure (from 50 to 400 MPa for 20–100 min). Similarly, Malone et al. [40] in a study about HHP effects (100–800 MPa, 5 min, 25 °C) on the activity of proteolytic and glycolytic enzymes, observed that plasmin was insensible to pressure treatments and the chymosin activity was unaffected by treatments up to 400 MPa, decreasing by 50% after an 800 MPa treatment. On the other hand, Juan et al. [28] found in ewes’ milk cheese that the chymosin activity decreases depending on the age of the cheese and the pressure applied (above 400 MPa, 1-day old cheeses), whereas plasmin activity was not significantly affected by HHP treatments (200–500 MPa, for 10 min) applied on the 1st and 15th day of ripening.

On ewes’ milk cheeses HHP15 samples showed slightly higher values on the three-proteolysis index evaluated than control samples during the ripening period, although no significant differences were observed at the end of the ripening, reflecting that this treatment did not significantly affect the proteolysis process. However, the HHP application during the initial stages of the ripening in ovine and caprine milk cheeses led to a decrease of the proteolysis rate. Similar results were obtained by Juan et al. [16] who in pressurized ovine milk cheeses on the 15th day of ripening obtained similar *WSN*/*TN* values than control samples, at the end of the ripening but higher than those obtained in samples with HHP-treatment on the 1st day. In contrast, some works reported after application of HHP treatments not differences on proteolysis indexes during ripening in Gouda [39] and cheddar cheeses [32] or an increase of proteolysis in goat milk cheese [41,42]. Starter bacteria are one of the primary sources of ripening intracellular enzymes (proteinases and peptidases). Cellular lysis is required for their release in the cheese matrix [43], being one of the main factors that influence the rate of secondary proteolysis [38]. HHP-induced cell lysis is pressure and strain-dependent [44] and possibly ripening time-HHP dependent. While Messens et al. [39] indicated that, in Gouda cheese, the possible lysis of the starter bacterial cells resulting from the damage suffered at 400 MPa did not increase proteolysis because endocellular enzymes were inactivated by the pressure, O’Reilly et al. [45] pointed out that ripening enzymes in cheddar cheese would probably begin to denature after the application of pressure treatments between 350–400 MPa and Juan et al. [16] and Calzada et al. [30] noticed that HHP-treatments above 400 MPa delay the proteolysis in ewes’ and cows’ milk, respectively. On the other hand, Saldo et al. [17,42] suggested that in goats’ milk cheeses, the release of starter enzymes probably caused an increase in proteolysis two weeks after HHP treatment at 400 MPa for 5 min was applied.

### 3.3. Effect of HHP on the Formation of BA on Cheeses

Table 5 and Table 6 show the values of the total BA content of the goats’ and ewes’ milk cheeses after HHP treatments. In general, the sum of all BA, including polyamines, showed a constant and significant increase in control samples during ripening from the 5th (59.36 mg/kg) to the 60th day (1156 mg/kg) of ripening in the goats’ milk cheese and from the 6th (85.08 mg/kg) to the 60th day (622.39 mg/kg) in ewes’ milk cheese samples. This is in agreement with the results reported by other authors [46,47,48,49,50,51]. The application of pressure during the first stages of ripening (HHP1) caused an initial reduction of BA formation from the 15th day, observing that at the end of the ripening these samples displayed concentrations around 75% lower than control samples. HHP15 ewes’ milk cheeses also resulted in a decrease of the BA content with respect to control samples, although this reduction was less pronounced (38% lower than control ones). The BA reduction in the HHP1 ewes’ and goats’ milk cheeses could be explained because of the significant decrease on microbiological counts observed one day after the treatment (specially of LAB, enteroccocci and enterobacteria) and the lower proteolysis presented in these samples with respect to the control ones, showing a reduction of about 34% and 49% of FAA content, respectively, at the end of the ripening. HHP15 samples also showed lower microbial counts when compared with the untreated cheeses but this did not affect the proteolysis and consequently the release of amino acids. Novella-Rodriguez et al. [52] found that the total BA content found in goats’ milk cheeses HHP treated at 400 MPa during 5 min was similar than the untreated cheeses, although TY content was significantly reduced in HHP samples. Ruiz-Capillas et al. [53] observed that HHP treatments at 350 MPa for 15 min used to treat “Chorizo” slices caused a significant decrease in BA content (TY, PU, CA and SM), being the reduction of these amines coincidental with the decrease in microbial counts, especially of LAB.

TY and PU were the main BA formed in untreated goats’ milk cheeses, showing concentrations of about 492 and 476 mg/kg, respectively, at the end of the ripening. Whereas in ewes’ milk control samples the predominant BA were TY and CA with 277 and 106 mg/kg, respectively. Several authors reported, in variable ranges, TY (88.6–445 mg/kg), HIS (not detected–697 mg/kg), PU (74.15–446.5 mg/kg) and CA (44–269.77 mg/kg) as the most abundant BA in goats’ and ewes’ milk ripened cheeses [26,46,48,49,54,55,56]. These variable contents depended on the type of cheese, length of the ripening period, the manufacturing process and the type of microorganisms present (starters and non-starter bacteria with decarboxylase activity). Post-ripening processing (cutting, slicing and grating) also has an important influence on the presence of decarboxylating bacteria in cheese and the formation of BA, such as HIS, may be greater than in entire cheeses [57]. TY levels in pressurized cheeses were lower than those presented in control cheeses. This aromatic amine increased slowly until the 15th day of ripening on goats’ milk HHP1 samples and remained constant throughout the rest of the ripening. At the end of the ripening the concentrations were about 93% lower than in control samples. Likewise, ewes’ milk HHP1 samples reached concentrations of 33 mg/kg at the 60th day, being 88% lower than control samples. The application of HHP treatment on the 15th day of ripening resulted in a slight decrease of TY levels in ewes’ milk cheeses and the final content was not significantly different than control samples at the 60th day of ripening. The reduction of the TY levels in pressurized goats’ and ewes’ milk cheese samples coincided mainly with the decrease in LAB and enteroccocci counts and with the amount of FAA. Novella-Rodriguez et al. [52] observed HHP-treated goats’ milk cheeses showed a significant lower TY content than untreated samples, attributing this behaviour to the reducing effect of HHP on the microbiological counts, especially on the non- starter LAB, although levels of TY in this kind of cheeses were similar. High “in vitro” capability to form TY has been described in different species of *Enterococcus* spp. and LAB isolated from cheese samples [12,58,59,60,61].

PU amounts in control goats’ milk cheeses were almost the same than TY but HHP1-treatments caused a decrease on the first 15 days, remaining almost stable throughout the rest of the ripening. PU levels were about 83% lower in the HHP1 samples than in control samples at the 60th day. The pressure application in ewes’ milk cheeses also affected the PU amounts formed, showing that HHP1 treatments limited the production of this diamine around 93% compared with control cheeses at the end of the ripening. HHP15 showed to be less efficient reducing the formation of PU. With respect to CA, untreated and HHP-treated goats’ milk cheeses displayed amounts below 100 mg/kg without appreciate significant differences between them. In control ewes’ milk cheeses this diamine increased mainly during the first 15 days, while in HHPI and HHP15 cheeses remained without significant changes throughout the ripening. The amounts of TR and PHE increased during the ripening in control goats’ milk cheese samples without significant differences in relation to HHP1-treated samples, while low amounts were detected in ewes’ cheeses remaining practically constant during ripening and without showing significant differences between treatments. Formation of PU and CA is usually associated with Gram negative bacteria, although some strains of *Enterococcus* spp. also have shown this capability “in vitro” [61]. Some LAB, such as *Lactococcus lactis*, are also able to form PU via the agmatine deiminase [62]. The application of the HHP treatments in the early stages of maturation causes an important reduction of both the Gram-negative microbiota, which practically disappeared and the enterococci that could not recover their initial counts during the rest of the ripening, which would explain that both PU and CA, together with TY, are the AB that present the greatest reduction. When treatments are applied after 15 days of maturation, the decarboxylating capacity of these microorganisms in the early stages of maturation is still present and consequently there would be a lesser effect on the formation of these AB. LAB are also affected by the HHP treatments, although later they are able to recover their initial counts, which is important to develop the cheese’s own characteristics, although they may also be responsible for the residual decarboxylating activity [30].

In goats’ raw milk control cheeses the concentration of HIS was very low at the beginning of ripening but increased later reaching its maximum after 45 days (18 mg/kg). No significant changes were observed until the 60th day. In HHP1-treated samples HIS showed a similar behaviour but in this case, differences were found after the 45th day displaying levels 68% lower than control samples at the 60th day of ripening. HHP1 ewes’ milk cheese samples showed a reduction of 92% of HIS when compared with control samples. This treatment was more efficient than the HHP15. Diverse authors reported low HIS amounts in cheese (below 100 mg/kg), relating the production of this BA with some LAB [12,31,54,56,63,64]. Some works have also described *Enterobacteriaceae* strains able to decarboxylate histidine in diverse foodstuffs [12,58,65,66,67,68,69]. Enterococci have also been related with histamine formation in cheeses [48,59,70].

In cheeses made from raw goat milk, low levels of polyamines were found at the beginning of ripening, increasing their concentration very slightly during ripening until the 60th day (Table 7). However, they showed an increase in their amount when HHP1- treatment was applied. Higher levels of SD were observed in ewes’ milk cheeses showing maximum amounts the 30th day of ripening (about 64 mg/kg in control cheeses) followed by a decrease at the end of the ripening. However, HHP1 treatment caused a significant increase of this polyamine at the 30th day, reaching maximum levels of about 122 mg/kg. SM was detected at constant amounts throughout the ripening in both control and HHP treated samples. SD has been reported as the main polyamine in some cheeses [10,46,55]. Novella-Rodríguez et al. [26] found in HHP treated goat cheeses an increase of polyamines, especially SD but no data were reported to elucidate the cause of this phenomenon. Polyamines are described as natural amines of non-microbial origin present generally at a lower concentration than other BA of bacterial origin. No toxic effects have been attributed to them although some authors have mentioned their importance for the intestine cell growth and proliferation in childhood [3,71].

## 4. Conclusions

The effectiveness of HHP treatments depends on different factors, such as the type of cheese, the stage of ripening, the HHP processing conditions applied, the kind and number of microorganisms present. In this work, the use of HHP applied at the initial phases of ripening affected significantly the microorganisms responsible of forming BA, and, in consequence, reduced its content, especially of TY and PU in goats’ milk cheeses and TY and CA in ewes’ milk cheeses, assuring the safety of this product for the most BA sensitive consumers. As previously mentioned, HIS and TY are the BA that most frequently have been related with food-borne outbreaks, being suggested threshold values in cheese between 200–400 mg/kg of HIS [6,72,73] and between 100–800 mg kg of TY [73,74]. TY dose of 6 mg [6] or above 20 mg of HIS [75] have been suggested as toxic to very sensitive individuals. If we consider the consumption of 30 g of cheese as a serving the goats’ and ewes’ cheeses analysed in this work would provide around 15 and 8.3 mg of TY, respectively, that can be dangerous for the most sensible consumers but HHP1-treated cheeses would provide with less than 1 mg of TY considering the same size of serving.

## Figures and Tables

**Table 1:** Changes in microbial population (mean values ± standard deviation as log_10_ CFU/g) of the goats’ raw milk cheese samples with and without high hydrostatic pressure (HHP) treatment during ripening.

Microbial Group	Day of Ripening	Control	HHP1
Lactococci	5	9.55 ± 0.27 ^a,A^	7.30 ± 0.22 ^B^
	15	8.75 ± 0.34 ^b,A^	6.76 ±0.49 ^B^
	30	8.47 ± 0.26 ^b,A^	7.03 ± 0.64 ^B^
	45	8.26 ± 0.20 ^b,A^	7.19 ± 0.25 ^B^
	60	7.54 ± 0.51 ^c,A^	7.05 ± 0.17 ^A^
Lactobacilli	5	6.54 ± 0.31 ^a,A^	3.13 ± 0.26 ^a,B^
	15	8.36 ± 0.42 ^b,A^	5.23 ± 1.54 ^b,B^
	30	8.28 ± 0.15 ^b,A^	6.31 ± 0.33 ^c,B^
	45	8.18 ± 0.19 ^b,A^	6.80 ± 0.50 ^c,B^
	60	7.89 ± 0.45 ^b,A^	7.03 ± 0.22 ^c,A^
Enterococci	5	6.23 ± 0.24 ^a,A^	4.44 ± 0.44 ^ab,B^
	15	6.10 ± 0.70 ^a,A^	3.99 ± 0.24 ^ab,B^
	30	6.17 ± 0.47 ^a,A^	4.60 ± 0.96 ^b,B^
	45	6.17 ± 0.48 ^a,A^	3.45 ± 0.34 ^a,B^
	60	5.32 ± 0.64 ^a,A^	3.85 ± 0.59 ^ab,B^
Enterobacteria	5	6.33 ± 0.26 ^a,A^	1.92 ± 0.54 ^a,B^
	15	3.15 ± 2.75 ^b,A^	2.49 ± 0.36 ^a,A^
	30	4.54 ± 0.15 ^b,A^	0.86 ± 1.48 ^a,B^
	45	3.58 ± 0.40 ^b,A^	ND ^b,B^
	60	2.94 ± 0.54 ^b,A^	ND ^b,B^
*E. coli*	5	2.56 ± 0.62 ^b,A^	ND ^B^
	15	0.90 ± 0.78 ^ab,A^	ND ^A^
	30	1.60 ± 1.96 ^ab,A^	0.83 ± 1.44 ^A^
	45	0.77 ± 0.68 ^ab,A^	ND ^A^
	60	ND ^a,A^	ND ^A^
*S. aureus*	5	2.64 ± 0.15 ^a,A^	0.43 ± 0.75 ^a,B^
	15	1.06 ± 0.96 ^b,A^	ND ^A^
	30	1.18 ± 0.31 ^b,A^	ND ^B^
	45	0.77 ± 1.33 ^b,A^	0.33 ± 0.58 ^B^
	60	ND ^b,A^	ND ^A^

ND: not detected (<10 CFU/g); means with different superscript small letter differ significant (*p* < 0.05) in the same column for the same parameter and HHP treatment; means with different superscript capital letter differ significant (*p* < 0.05) in the same row for the same parameter and day of ripening; HHP1: HHP treated the 5th day of the ripening.

**Table 2:** Changes in microbial population (mean values ± standard deviation as log_10_ CFU/g) of the ewes’ raw milk cheese samples with and without HHP treatment during ripening.

Microbial Group	Day of Ripening	Control	HHP1	HHP15
Lactococci	5	9.34 ± 0.33 ^a,A^	7.80 ± 0.31 ^a,B^	
	15	9.16 ± 0.20 ^a,A^	6.87 ± 0.39 ^b,B^	7.25 ± 0.22 ^a,B^
	30	9.21 ± 0.18 ^a,A^	8.04 ± 0.74 ^b,B^	7.67 ± 0.14 ^ab,B^
	45	8.74 ± 0.20 ^ab,A^	8.07 ± 0.61 ^b,B^	7.92 ± 0.39 ^ab,B^
	60	8.38 ± 0.16 ^b,A^	8.32 ± 0.76 ^b,A^	7.57 ± 0.32 ^b,B^
Lactobacilli	5	5.57 ± 0.35 ^a,A^	4.54 ± 0.64 ^a,B^	
	15	7.52 ± 1.07 ^b,A^	5.60 ± 0.37 ^b,B^	6.19 ± 0.50 ^a,B^
	30	8.64 ± 0.41 ^c,A^	6.61 ± 0.82 ^c,B^	6.78 ± 1.00 ^ab,B^
	45	8.68 ± 0.12 ^c,A^	8.09 ± 0.55 ^d,A^	7.82 ± 0.27 ^bc,A^
	60	8.15 ± 0.39 ^c,A^	8.12 ± 0.51 ^d,A^	7.53 ± 0.15 ^c,B^
Enterococci	5	5.59 ± 0.74 ^A^	3.16 ± 1.05 ^A^	
	15	6.18 ± 2.14 ^AB^	3.50 ± 1.13 ^C^	3.90 ± 1.11 ^B^
	30	6.16 ± 1.18 ^A^	1.76 ± 1.53 ^B^	3.96 ± 1.32 ^A^
	45	4.63 ± 1.04 ^A^	2.76 ± 2.26 ^B^	3.27 ± 0.48 ^A^
	60	4.23 ± 1.40 ^A^	2.58 ± 1.93 ^B^	3.88 ± 0.93 ^A^
Enterobacteria	5	4.65 ± 0.23 ^a,A^	ND ^B^	
	15	4.46 ± 0.30 ^a,A^	0.29 ± 0.58 ^B^	ND ± ND ^B^
	30	4.42 ± 1.92 ^a,A^	ND ^B^	1.22 ± 1.11 ^B^
	45	2.87 ± 1.21 ^b,A^	ND ^B^	0.57 ± 0.98 ^B^
	60	2.35 ± 0.96 ^b,A^	ND ^B^	ND ^B^
*E. coli*	5	1.16 ± 1.01	ND	
	15	1.15 ± 1.37	ND	ND
	30	0.65 ± 0.79	ND	ND
	45	0.64 ± 0.73	ND	0.78 ± 1.35
	60	0.73 ± 0.91	ND	ND
*S. aureus*	5	2.35 ± 0.58 ^a^	1.07 ± 1.35	
	15	1.74 ± 1.19 ^ab^	0.50 ± 0.58	0.33 ± 0.58
	30	1.17 ± 0.35 ^ab^	0.29 ± 0.58	0.33 ± 0.58
	45	0.61 ± 0.71 ^bc^	ND	ND
	60	0.25 ± 0.50 ^c^	ND	ND

ND: Not detected (<10 CFU/g); means with different superscript small letter differ significant (*p* < 0.05) in the same column for the same parameter and HHP treatment; means with different superscript capital letter differ significant (*p* < 0.05) in the same row for the same parameter and day of ripening; HHP1: HHP treated the 5th day of the ripening; HHP15: HHP treated after 15 days of ripening.

**Table 3:** Changes on proteolysis index (mean values ± standard deviation) during the ripening of goats’ raw milk cheese samples with and without HHP treatment (TNBS: amino group content determined by the Trinitrobenzensulphonic Acid method, RI: Ripening Index, FFA: free Amino Acid content; TNBS and FAA expressed in mg l-Leucine/g).

Proteolysis Index	Day of Ripening	Control	HHP1
TNBS	5	10.40 ± 2.24 ^a^	9.41 ± 1.80 ^a^
	15	14.90 ± 1.21 ^ab^	15.33 ± 2.15 ^ab^
	30	22.54 ± 2.50 ^b^	21.14 ± 4.59 ^b^
	45	44.89 ± 1.19 ^c,B^	32.65 ± 5.81 ^c,A^
	60	53.66 ± 2.14 ^d,B^	39.96 ± 4.52 ^c,A^
RI	5	10.99 ± 3.05 ^a^	11.09 ± 2.60 ^a^
	15	22.97 ± 1.23 ^b^	22.97 ± 1.23 ^b^
	30	21.88 ± 0.60 ^b^	24.39 ± 3.15 ^bc^
	45	22.14 ± 1.78 ^b^	25.71 ± 2.15 ^bc^
	60	25.07 ± 0.66 ^b^	28.03 ± 0.35 ^c^
FAA	5	2.08 ± 0.57 ^a^	1.48 ± 0.72 ^a^
	15	4.85 ± 1.42 ^a^	3.71 ± 1.06 ^ab^
	30	10.05 ± 2.06 ^b,B^	5.90 ± 1.59 ^bc,A^
	45	15.07 ± 3.52 ^c,B^	8.40 ± 1.43 ^cd,A^
	60	15.71 ± 3.06 ^c,B^	10.40 ± 1.29 ^d,A^

Means with different superscript small letter differ significant (*p* < 0.05) in the same column for the same parameter and HHP treatment; means with different superscript capital letter differ significant (*p* < 0.05) in the same row for the same parameter and day of ripening.

**Table 4:** Evolution of proteolysis index (mean values ± standard deviation) during the ripening of ewes’ raw milk cheese samples with and without HHP treatment.

Proteolysis Index	Day of Ripening	Control	HHP1	HHP15
TNBS	5	7.66 ± 1.65 ^a^	7.92 ± 0.44 ^a^	
	15	13.87 ± 1.37 ^ab^	13.36 ± 0.82 ^a^	15.03 ± 0.40 ^a^
	30	22.13 ± 8.22 ^b,AB^	26.33 ± 17.48 ^b,BC^	34.31 ± 6.11 ^b,C^
	45	34.93 ± 2.60 ^c,BC^	28.00 ± 2.84 ^b,AB^	44.79 ± 4.93 ^bc,C^
	60	47.40 ± 2.74 ^d,BC^	37.59 ± 6.86 ^b,A^	52.50 ± 2.21 ^c,C^
RI	5	9.70 ± 3.55 ^a^	10.44 ± 4.39 ^a^	
	15	19.38 ± 4.24 ^b^	18.51 ± 4.23 ^b^	19.15 ± 2.57 ^a^
	30	24.96 ± 3.64 ^bc^	23.35 ± 2.38 ^b^	25.39 ± 3.57 ^ab^
	45	29.59 ± 2.97 ^c,B^	25.09 ± 2.95 ^b,A^	30.54 ± 2.46 ^b,B^
	60	31.92 ± 6.63 ^c,B^	26.38 ± 3.77 ^b,A^	31.62 ± 2.21 ^b,B^
FAA	5	2.36 ± 0.04 ^a^	2.18 ± 0.22 ^a^	
	15	5.01 ± 1.27 ^a^	3.66 ± 1.79 ^ab^	5.59 ± 2.94 ^a^
	30	11.66 ± 2.76 ^b,B^	6.85 ± 3.07 ^ab,AB^	11.22 ± 1.11 ^b,B^
	45	11.39 ± 2.69 ^b,B^	6.55 ± 1.10 ^ab,AB^	14.86 ± 4.89 ^bc,C^
	60	16.56 ± 3.51 ^c,B^	8.63 ± 1.71 ^b,A^	16.03 ± 5.14 ^c,B^

Means with different superscript small letter differ significant (*p* < 0.05) in the same column for the same parameter and HHP treatment; means with different superscript capital letter differ significant (*p* < 0.05) in the same row for the same parameter and day of ripening; TNBS: amino group content determined by the Trinitrobenzensulphonic Acid method, RI: Ripening Index, FFA: free Amino Acid content; TNBS and FAA expressed in mg l-Leucine/g.

**Table 5:** Monoamine and diamines content (mean values ± standard deviation expressed as mg/kg in dry basis) formed during the ripening of goats’ raw milk cheese samples with and without HHP treatment.

BA	Day of Ripening	Control	HHP1
TR	5	8.99 ± 7.94 ^a^	23.12 ± 5.93 ^a^
	15	27.76 ± 15.00 ^ab^	10.81 ± 9.37 ^a^
	30	95.96 ± 34.38 ^c^	82.10 ± 26.33 ^b^
	45	68.39 ± 20.42 ^bc^	70.11 ± 40.01 ^b^
	60	63.69 ± 27.90 ^bc^	89.08 ± 49.98 ^b^
PHE	5	1.40 ± 0.65 ^a^	0.26 ± 0.45 ^a^
	15	14.62 ± 1.42 ^b^	17.96 ± 4.07 ^b^
	30	17.27 ± 5.94 ^b^	17.16 ± 4.29 ^b^
	45	20.75 ± 4.20 ^ab^	14.80 ± 0.45 ^ab^
	60	31.13 ± 14.19 ^b^	25.56 ± 7.47 ^b^
PU	5	4.07 ± 0.51 ^a^	3.29 ± 0.42
	15	136.56 ± 21.57 ^ab^	59.09 ± 7.34
	30	225.16 ± 22.31 ^b^	67.09 ± 8.50
	45	463.88 ± 60.73 ^c,B^	42.69 ± 33.89 ^A^
	60	476.41 ± 126.21 ^c,B^	79.80 ± 19.51 ^A^
CA	5	30.20 ± 16.19 ^a^	24.07 ± 9.04
	15	29.63 ± 4.35 ^a^	26.20 ± 3.13
	30	50.14 ± 10.97 ^ab^	35.29 ± 12.70
	45	69.53 ± 16.34 ^b^	36.22 ± 17.00
	60	70.45 ± 27.21 ^b^	44.22 ± 15.61
HIS	5	1.27 ± 0.56 ^a^	1.00 ± 0.87
	15	3.02 ± 0.43 ^a^	2.44 ± 0.34
	30	6.38 ± 3.31 ^a^	6.27 ± 3.16
	45	18.04 ± 9.36 ^b,B^	6.51 ± 1.75 ^A^
	60	15.41 ± 7.05 ^b,B^	4.85 ± 2.20 ^A^
TY	5	10.04 ± 6.80 ^a^	6.11 ± 6.95
	15	130.51 ± 42.98 ^ab^	18.96 ± 1.07
	30	234.74 ± 69.16 ^b,B^	15.59 ± 3.56 ^A^
	45	443.87 ± 105.10 ^c,B^	16.17 ± 0.68 ^A^
	60	491.89 ± 67.45 ^c,B^	28.93 ± 5.91 ^A^

Means with different superscript small letter differ significant (*p* < 0.05) in the same column for the same parameter and HHP treatment; means with different superscript capital letter differ significant (*p* < 0.05) in the same row for the same parameter and day of ripening. TR: tryptamine, PHE: β-phenylethylamine, PU: putrescine, CA: cadaverine, HIS: histamine, TY: tyramine.

**Table 6:** Monoamine and diamines content (mean values ± standard deviation expressed as mg/kg in dry basis) formed during the ripening of ewes’ raw milk cheese samples with and without HHP treatment.

BA	Day of Ripening	Control	HHP1	HHP15
TR	5	1.66 ± 3.31 ^a^	4.92 ± 5.86	-
	15	4.51 ± 3.32 ^a^	10.03 ± 7.29	8.76 ± 6.98
	30	9.87 ± 4.43 ^ab^	9.83 ± 4.66	6.25 ± 0.80
	45	9.51 ± 3.85 ^ab^	12.49 ± 6.76	12.32 ± 8.43
	60	15.73 ± 2.15 ^b^	11.06 ± 6.78	11.66 ± 6.50
PHE	5	1.02 ± 1.23 ^a^	0.40 ± 0.48	-
	15	2.78 ± 1.44 ^a^	2.39 ± 0.56	4.39 ± 1.84
	30	5.67 ± 5.25 ^a^	3.04 ± 1.01	3.71 ± 1.45
	45	12.69 ± 5.08 ^ab^	4.37 ± 1.52	5.78 ± 1.53
	60	12.74 ± 2.62 ^b^	4.43 ± 2.37	13.31 ± 15.37
PU	5	3.62 ± 0.40 ^a^	5.20 ± 2.41	-
	15	42.42 ± 23.00 ^ab^	11.25 ± 9.80	15.14 ± 11.99
	30	65.89 ± 30.90 ^bc,B^	5.45 ± 1.38 ^A^	22.33 ± 14.49 ^AB^
	45	87.89 ± 76.10 ^c,B^	7.81 ± 3.03 ^A^	22.92 ± 17.51 ^A^
	60	74.89 ± 54.74 ^bc,B^	5.24 ± 0.76 ^A^	26.08 ± 6.26 ^A^
CA	5	62.13 ± 33.00 ^a^	55.55 ± 28.54	-
	15	159.07 ± 37.23 ^b,B^	48.75 ± 44.16 ^A^	87.50 ± 70.43 ^AB^
	30	141.63 ± 79.34 ^ab,B^	48.40 ± 16.94 ^A^	80.65 ± 76.20 ^AB^
	45	129.82 ± 67.03 ^ab,B^	41.91 ± 22.89 ^A^	72.47 ± 29.78 ^AB^
	60	105.90 ± 21.87 ^ab,B^	28.51 ± 17.32 ^A^	80.28 ± 71.97 ^AB^
HIS	5	4.81 ± 4.54 ^a^	5.33 ± 3.79	-
	15	39.98 ± 11.42 ^b^	12.71 ± 10.97	29.14 ± 22.84 ^a^
	30	74.34 ± 29.42 ^c,B^	6.38 ± 2.77 ^A^	47.52 ± 31.34 ^ab,B^
	45	81.66 ± 46.30 ^c,B^	5.94 ± 1.25 ^A^	58.99 ± 3.14 ^b,B^
	60	91.02 ± 5.73 ^c,C^	7.07 ± 4.30 ^A^	57.65 ± 13.16 ^ab,B^
TY	5	4.37 ± 0.71 ^a^	3.15 ± 1.94 ^a^	-
	15	123.72 ± 40.62 ^ab,B^	13.79 ± 5.28 ^b,A^	115.79 ± 55.44 ^B^
	30	222.32 ± 84.26 ^bc,B^	22.16 ± 10.34 ^b,A^	162.64 ± 36.21 ^A^
	45	268.02 ± 130.91 ^c,B^	80.49 ± 126.16 ^ab,A^	147.73 ± 30.57 ^AB^
	60	277.30 ± 114.08 ^c,B^	32.69 ± 16.54 ^b,A^	147.62 ± 26.64 ^AB^

Means with different superscript small letter differ significant (*p* < 0.05) in the same column for the same parameter and HHP treatment; means with different superscript capital letter differ significant (*p* < 0.05) in the same row for the same parameter and day of ripening. TR: tryptamine, PHE: β-phenylethylamine, PU: putrescine, CA: cadaverine, HIS: histamine, TY: tyramine.

**Table 7:** Polyamine content (mean values ± standard deviation expressed as mg/kg in dry basis) during the ripening of goats’ and ewes’ raw milk cheese samples with and without HHP treatment. (SD: spermidine, SM: spermine).

	Day of Ripening	Control	HHP1	HHP15
**Goats’ milk chesses**				
SD	5	1.13 ± 0.51 ^a^	0.32 ± 0.50 ^a^	-
	15	1.92 ± 0.18 ^aA^	4.34 ± 0.21 ^b,B^	-
	30	2.09 ± 0.24 ^abA^	3.70 ± 0.89 ^b,B^	-
	45	4.32 ± 1.75 ^c^	3.28 ± 1.30 ^b^	-
	60	3.92 ± 1.49 ^bcA^	6.53 ± 1.99 ^c,B^	-
SM	5	2.25 ± 2.07 ^a^	1.47 ± 1.48 ^a^	-
	15	4.35 ± 1.25 ^ab,A^	7.80 ± 0.60 ^bc,B^	-
	30	3.03 ± 0.75 ^ab,A^	6.83 ± 0.46 ^b,B^	-
	45	5.57 ± 3.91 ^b,B^	2.16 ± 0.30 ^a,A^	-
	60	3.90 ± 0.75 ^ab,A^	10.46 ± 2.18 ^c,B^	-
**Ewes’ milk cheeses**				
SD	5	0.73 ± 0.65 ^a^	0.51 ± 0.90 ^a^	-
	15	55.94 ± 29.00 ^ab^	90.23 ± 67.99 ^bc^	73.15 ± 38.57 ^b^
	30	64.22 ± 8.22 ^b,A^	122.25 ± 48.16 ^c,B^	65.61 ± 10.97 ^b,A^
	45	14.74 ± 14.76 ^ab^	52.04 ± 36.91 ^ab^	34.49 ± 19.56 ^ab^
	60	30.14 ± 23.04 ^ab^	19.49 ± 33.57 ^a^	17.36 ± 9.35 ^a^
SM	5	6.76 ± 5.17	5.94 ± 4.03 ^a^	
	15	14.13 ± 12.84	18.69 ± 14.57 ^a^	8.00 ± 6.99 ^a^
	30	25.18 ± 18.41	28.39 ± 7.52 ^b^	39.50 ± 32.16 ^b^
	45	17.57 ± 9.32	13.13 ± 7.26 ^ab^	20.08 ± 21.91 ^ab^
	60	14.66 ± 17.02	15.27 ± 17.76 ^a^	26.92 ± 17.93 ^ab^

Means with different superscript small letter differ significant (*p* < 0.05) in the same column for the same parameter and HHP treatment; means with different superscript capital letter differ significant (*p* < 0.05) in the same row for the same parameter and day of ripening.
